# Ultrasound examination of the teat parameters of mastitis and healed udder quarters

**DOI:** 10.1016/j.vas.2023.100296

**Published:** 2023-05-22

**Authors:** Tamás Tóth, Miklós Tamás Tóth, Zsolt Abonyi-Tóth, Vanessa Silva, Patricia Poeta, Mihály Sipos, Alexandra Juhász

**Affiliations:** a Ruminant Health Service, Budapest H-1044, Hungary; b Gulner Gyula School, Budapest H-1183, Hungary; c Department of Biostatistics, University of Veterinary Science, Budapest H-1078, Hungary; d LAQV-REQUIMTE, Department of Chemistry, NOVA School of Science and Technology, Universidade Nova de Lisboa, Caparica 2829-516, Portugal; e CECAV—Veterinary and Animal Research Centre, University of Trás-os-Montes and Alto Douro (UTAD), Vila Real 5000-801, Portugal; f Associate Laboratory for Animal and Veterinary Science (AL4AnimalS), University of Trás-os-Montes and Alto Douro (UTAD), Vila Real 5000-801, Portugal; g Tulip Farm, Cegléd H-2600, Hungary; h Department of Tropical Disease Biology, Liverpool School of Tropical Medicine, Liverpool L3 5QA, UK; i Institute of Medical Microbiology, Semmelweis University, Budapest H-1089, Hungary

**Keywords:** Cattle, Mastitis, Milking, Teat, Ultrasound

## Abstract

A healthy udder is important for economical and hygienic milk production. In this study we determined the teat parameters, measured during milking, in the presence of mastitis and after its recovery. The length of the streak canal, the area of the teat end and the area of the pars papillaris of 52 Holstein–Friesian cows were examined by ultrasound before, immediately after, and two hours after milking. Pars papillaris size before milking was higher in the presence of disease (P = 0.011), but was larger before and two hours after milking (P = 0.047 and P = 0.024) with more severe mastitis. During mastitis and recovery, the dimensions of the streak canal (P=0.056–0.934) and teat end (P=0.156–0.420) did not differ from each other. The obtained results show that during mastitis, coagulated milk dilates the pars papillaris better (P=0.011) than the higher milk production of the cured cow. After more severe mastitis, regeneration of the pars papillaris as well as post-milking regeneration also takes place more slowly. The size and size changes of the streak canal and teat end are not affected by the presence or healing of the udder inflammation.

## Highlights

• In mastitis, coagulated milk significantly expands (P = 0.011) the pars papillaris.
• After more severe mastitis, the regeneration of the pars papillaris is slower.
• Mastitis and its healing have no effect on the size of the streak canal and teat end.

## Graphical abstract

**Figure:**
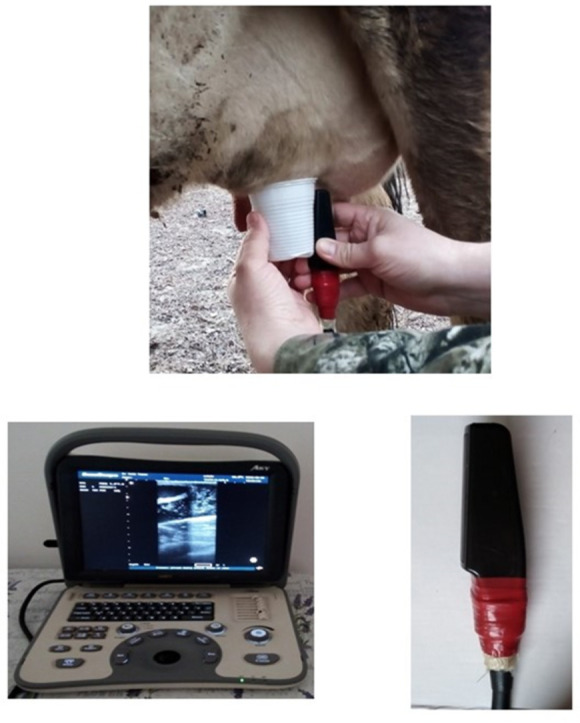

## 1 Introduction

Mastitis is an economically important pathology associated with costs, 5–7% annual turnover of dairy farms (Mekonnen et al., 2019; Ózsvári, 2012). These costs are related to veterinary treatments, excess labour demand, premature culling, discarded milk, long-term production interruptions and decreased milk quality (Heikkilä et al., 2012). Pathogens most often enter the udder *via* the streak canal (Murphy, 1944). This infection is usually prevented by the structure of the tip of the teat. The anatomical structure and function of the teat end can be thoroughly examined by ultrasound. In previous research several parameters of the teat end have been examined, these include the length and thickness of the streak canal (Fasulkov et al., 2014; Strapák et al., 2017; Porter, Wieland, & Basran, 2021), the thickness of the teat wall (Stádnik et al., 2010), the area of the teat end (Porter et al., 2021Porter et al., 2021), the diameter of the pars papillaris (Stojnovič & Alagič, 2012) and the thickness of the teat (Klein et al., 2005). The authors examined the influence of age (Celik et al., 2008), breed (Klein et al., 2005; Seker et al., 2009), lactation number (Szencziová et al., 2013), milk yield (Comalli et al., 1984) or milking (Porter et al., 2021) on certain anatomical parts of the teat on healthy animals.

When comparing the teats of healthy cows and cows with mastitis, Klein et al. (2005) found that the length of the streak canal (P=0.002) was larger in healthy teats, while its diameter (P=0.012) was smaller than that of diseased teats. also found no significant (P>0.01) difference between the different degrees of mastitis in the length of the streak canal, teat diameter measured at the height of Fürstenberg's rosette, and teat wall thickness. In contrast, Hamana et al. (1994) found that the length of the streak canal was not different between the teat of healthy and sick cows, but the diameter of the streak canal was significantly (P=0.0211) larger than that of healthy cows.

In previous publications, the teat parameters of diseased cows were always compared with those of healthy cows. In our research, however, we examined the size of the teat parameters in the case of a specific cow during mastitis and after healed.

In the course of our research, three teat parameters, the length of the streak canal, the area of the teat tip and the 1 cm area of the pars papillaris distalis were examined by ultrasound. In connection with mastitis, we examined the size changes of these three eat parameters as a result of milking.

During our research, we set the following goals:

1 To determine to what extent the size of the 3 teat parameters measured during milking in the presence of mastitis differs from the values measured after healed.
2 To determine to what extent the size of the 3 teat parameters measured during milking in the presence of mastitis differs for cows with different degrees of mastitis.
3 To determine to what extent the size of the 3 teat parameters measured during milking and after the mastitis has healed differs in the case of cows with different degrees of mastitis.

## 2 Material and methods

### 2.1 Study area and design

Our study was conducted in a dairy cattle farm in Veszprém county in Hungary. The average number of cows in the cattle farm is 876, the annual milk production is 8969 kg / cow. The cows were milked twice a day in a 2 × 24-position milking parlour by a Westfalia-type milking machine (vacuum size: 42 KPa, pulsation ratio: 60:40, pulsation rate: 62).

Cows were tested using the California Mastitis Test (CMT) before morning milking and cows with a positive CMT result (N=52) were included in the study.

Based on the results of the CMT, we divided the cows into two roups:

Group 1 : cows with mastitis degree 2+ (26 cows)

Group 2 : cows with mastitis degree 3+ (26 cows)

The cows participating in the study were in their 3rd lactation.

The ultrasound examinations were performed three times during the morning milking (before milking, immediately after milking and 2 h after milking).

The cows participating in our research were examined twice: the first examination was performed when mastitis was diagnosed, when the CMT was positive, and the second examination was performed after the mastitis had healed and the CMT was negative.

In accordance with our objective, the values measured during the diagnosis of the inflammation and after recovery were as follows:

1 The values measured during milking of sick cows were compared with the values measured after recovery.
2 We compared the existing measured values of mastitis of cows with mastitis degree 2+ and 3+.
3 We compared the following measured values of the healing of mastitis in cows with 2+ and 3+ degrees of mastitis.

### 2.2 Ultrasound examination

**Fig. 1:**
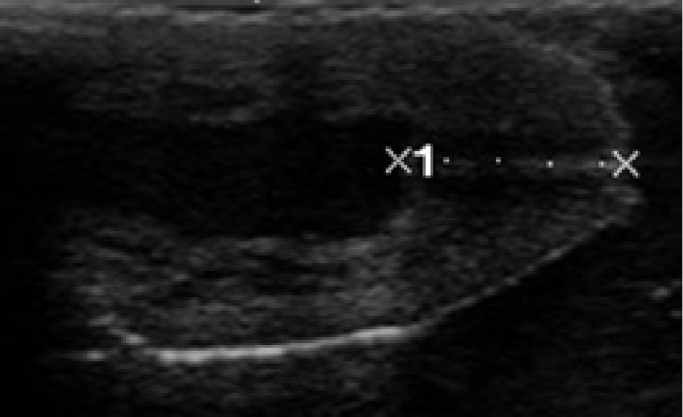
The lenght of the teat canal.

**Fig. 2:**
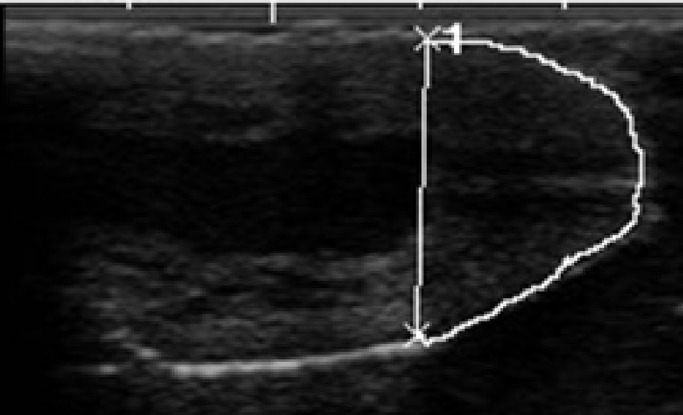
The area of the teat end.

**Fig. 3:**
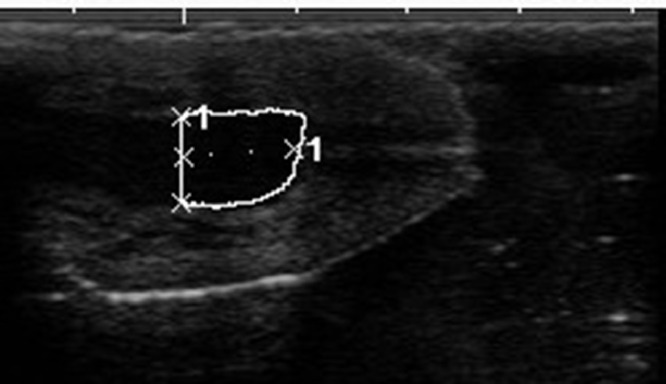
The distal 1 cm area of pars papillaris.

For the ultrasonic analysis a SonoScape A6 ultrasound machine and 5–7 MHz linear transducer were used. The teats of the cows were examined with a so called water bath method, in which the teats were immersed one by one in a 200 ml 35 °C water-filled plastic cup and the transducer applied with ultrasonic gel was placed outside of the plastic cup wall parallel to the teat's longitudinal axis. By this method the three parameters of the teat were measured. These are; the length of the streak canal-the distance between the outer and the inner opening of the streak canal in mm-(Fig. 1); the area of the teat end in cm² - (Fig. 2) and the distal 1 cm area of pars papillaris: up to a height of 1 cm measured proximal to the Fürstenberg rosette, the area of the pars papillaris in cm² (Fig. 3) were measured.

### 2.3 Data analysis

The statistical analysis was performed by SPSS-software version 18. The teat parameter values of cows at mastitis and after healing were compared with a paired t-test. Two-sample t-test was used to compare the length of the streak canal, the area of the teat end and area of pars papillaris between the cows which has 2+ or 3+ mastitis degree. The significance level was set at P <0.05.

## 3 Results

In mastitis, the values of the length of the teat canal and the area of the teat before (P=0.056 and P=0.156), after (P=0.57 and P=0.211) and two hoursafter (P=0.934 and P=0.42) milking did not differ significantly from those after recovery. However, a significant difference (P = 0.011) was found in the distal 1 cm area of the pars papillaris during the pre-milking measurement. The area of this part of the teat was on average 11.7% larger during the disease than after recovery (Table 1).

**Table 1:** 2. Comparison of teat parameter values measured at milking time during illness and after recovery.

**Time of measurement**	**Teat parameters**	**Difference in averages**	**Difference error SE**	P-value
**Before milking**	The length of streak canal	−0.523	1.833	0.056
	The area of pars papillaris	0.110	0.282	0.011*
	The area of teat end	−0.069	0.329	0.156
**After milking**	The length of streak canal	−0.209	2.499	0.570
	The area of pars papillaris	0.028	0.194	0.333
	The area of teat end	−0.093	0.504	0.211
**Two hours after milking**	The length of streak canal	0.028	2.288	0.934
	The area of pars papillaris	0.059	0.263	0.128
	The area of teat end	−0.052	0.441	0.420

⁎ P<0.05.

After CMT examination, the cows were divided into two groups based on the degree of mastitis. When comparing these two groups it was found that there was no significant difference (P>0.05) between the groups and any of the teat parameters.

The values of the teat parameter measured after healing in the udder quarters with 2+ and 3+ degrees of udder inflammation showed that the 1 cm area of the pars papillaris distal was significantly smaller (P = 0.047 and P = 0.024) in the pre-milking and 2 h measurements of pars papillaris in the udder quarters, which had a degree of inflammation of 2+ degrees. We did not find a significant difference between the two degrees of mastitis in the case of the length of the streak canal (P=0.896, P=0,978 and P=0.519) and the area of the teat end (P=0.929, P=0.905 and P=0.488) (Table 3).

## 4 Discussion

The average daily milk production of cows with mastitis was 13.7 kg, while after healing it was 24.6 kg. Thus, cows produced an average of 79.6% (P = 0.0001) more milk after healing than when infected. Previous literature data showed that there is a very close positive correlation (r=0.92) between the area of the milk cistern and pars papillaris measured by ultrasound and the amount of milk produced (Ayadi et al., 2003). Before the start of milking, only 30–40% of the milk is in the milk ducts and milk cistern, this means that the majority of the milk is precent in the pars papillaris before milking (Tóth & Bak, 1980). Based on the milk production data of our own study and previous literature data, we expected the result that the area of the pars papillaris of healed cows would be larger as a result of the increased milk production. In contrast, it was observed that during the existence of the disease, the pars papillaris measured before milking was significantly wider (P=0.011) than after recovery. This is due to the fact that during mastitis, milk coagulates in the milk ducts, and this coagulated milk expands the pars papillaris to a larger degree during inflammation than the larger amount of milk after healing.

No significant difference (P=0.203–0.848) was found between the teat parameters of 2+ and 3+ degrees of mastitis of udder quarters in the case presence of the disease. Our research findings are supported by test results of , according to which there was no significant (P> 0.01) difference between the different degrees of mastitis in the length of the streak canal (P=0.102), the diameter of the teat at the height of the Fürstenberg rosette (P=0.084), and the thickness of the teat wall (P=0.118). Previous research and our test results show that the degree of mastitis in cows with udder disease does not affect the anatomical dimensions of the teat parameters measured by ultrasound (Table 2).

**Table 2:** Comparison of teat parameters measured at milking time of cows with different degrees of mastitis when the disease exists.

**Time of measurement**	**Teat parameters**	**Difference in averages**	**Difference error SE**	P-value
**Before milking**	The length of streak canal	−0.669	0.752	0.378
	The area of pars papillaris	0.074	0.076	0.335
	The area of teat end	−0.122	0.123	0.326
**After milking**	The length of streak canal	−0.160	0.829	0.848
	The area of pars papillaris	−0.031	0.059	0.600
	The area of teat end	−0.047	0.148	0.750
**Two hours after milking**	The length of streak canal	−0.666	0.729	0.366
	The area of pars papillaris	0.106	0.082	0.203
	The area of teat end	−0.060	0.124	0.631

P<0.05.

**Table 3:** Comparison of teat parameters measured at milking time of cows with different degrees of mastitis after recovery.

**Time of measurement**	**Teat parameters**	**Difference in averages**	**Difference error SE**	P-value
**Before milking**	The length of streak canal	0.093	0.701	0.896
	The area of pars papillaris	0.156	0.076	0.047*
	The area of teat end	−0.012	0.128	0.929
**After milking**	The length of streak canal	0.027	0.993	0.978
	The area of pars papillaris	0.018	0.070	0.794
	The area of teat end	−0.023	0.189	0.905
**Two hours after milking**	The length of streak canal	−0.496	0.763	0.519
	The area of pars papillaris	0.145	0.062	0.024*
	The area of teat end	−0.091	0.131	0.488

⁎ P<0.05.

After mastitis healing, the pars papillaris area of 2+ mastitis cows measured before and two hours after milking was significantly (P = 0.047 and P = 0.024) smaller than that of 3+ cows. In the light of these results, we found that after the recovery of more severe mastitis, the area of the pars papillaris before milking is less pronounced than in the case of milder mastitis. Similarly, the severity of mastitis has an effect on the regeneration of size changes caused by milking, with the area of the pars papillaris at two hours post-milking (P = 0.024) higher in the udder quarter from more severe mastitis than in lighter mastitis cows. The size of the teat end and streak canal, which form an important part of the udder's defense system, was affected not only during the existence of the inflammation, but also by the inflammation following its healing.

From a clinical point of view, it is important that neither the existence of mastitis nor its healing affect the size and size change of the streak canal and teat end, which form the defense system of the udder. In line with our research results, did not find any significant (P>0.05) differences between sick and healthy cows when measuring the length of the streak canal. However, during Klein's experiment in 2005, contrary to our results, he found the streak canal of healthy cows to be significantly (P=0.012) longer.

## 5 Conclusions

During mastitis, the coagulated milk dilates pars papillaris better (P = 0.011) than higher milk production in a cured cow. After more severe mastitis, pars papillaris regeneration and post-milking regeneration are also slower. Neither the presence of the mastitis nor its healing is affected by the size and size change of the streak canal and the teat end, which is part of the udder defense system.

## Ethical statement

The authors assert that all procedures contributing to this work comply with the ethical standards of the relevant national and institutional guides on the care and use of vertebrates. No experiments on animals.

## Declaration of Competing Interest

The authors declare that they have no known competing financial interests or personal relationships that could have appeared to influence the work reported in this paper.
